# A Rare Case of Severe Manifestation of COVID-19 Infection Presenting as Immune-Related Thrombotic Thrombocytopenic Purpura With Multiorgan Involvement Treated With Plasmapheresis, Steroids, Rituximab, and Caplacizumab

**DOI:** 10.7759/cureus.26961

**Published:** 2022-07-18

**Authors:** Saikiran Mandyam, Syed S Fatmi, George Banzon, Paramjit Kaur, Yamini Katamreddy, Devam Parghi, Awais Farooq, Hamza Liaqat, Krishnamohan Basarakodu

**Affiliations:** 1 Internal Medicine, Southeast Health Medical Center, Dothan, USA; 2 Internal Medicine, Alabama College of Osteopathic Medicine, Dothan, USA; 3 Internal Medicine, West Anaheim Medical Center, Anaheim, USA; 4 Internal Medicine, Wah Medical College, Wah Cantonment, PAK; 5 Hematology and Oncology, Southeast Health Medical Center, Dothan, USA

**Keywords:** caplacizumab, rituximab, hemodialysis, plasmapheresis, adamts13 activity, adamts13 inhibitor levels, schistocytes, hemolytic anemia, thrombotic thrombocytopenic purpura

## Abstract

Thrombotic thrombocytopenic purpura (TTP) is a thrombotic microangiopathy (TMA) caused by decreased activity of a disintegrin and metalloproteinase with a thrombospondin type 1 motif, member 13 (ADAMTS13). Platelet-rich thrombi in small vessels lead to fragmentation of RBCs causing microangiopathic hemolytic anemia (MAHA). Therapeutic plasma exchange is life-saving and is the mainstay of the treatment of TTP. Higher dose IV steroids along with rituximab are used as an adjunct to plasma exchange.

Our case report describes a 26-year-old healthy male who presented with new onset seizures and encephalopathy. Blood work demonstrated anemia, severe thrombocytopenia, elevated lactate dehydrogenase, decreased haptoglobin, and elevated creatinine, and peripheral blood smear showed marked schistocytosis indicating MAHA. Plasma exchange and high-dose steroids were started on a presumptive diagnosis of TTP. ADAMTS13 activity was undetectable and ADAMTS13 inhibitor levels were elevated. Rituximab and caplacizumab were then added. Symptoms of encephalopathy improved by day five and platelet counts started improving by day nine. After several days of plasma exchange, he showed a “clinical response” with several weeks of active treatment. The association between coronavirus disease 2019 (COVID-19) infection and the severity of TTP with multiorgan failure is not well understood yet. Although we describe a successful multimodal approach to the management of TTP, which we believe is secondary to COVID-19 infection, further research is warranted to analyze and understand the pathophysiology by which COVID-19 infection causes TTP. It would help in establishing standardized therapy in the future.

## Introduction

Coronavirus disease 2019 (COVID-19) has been challenging with the predominance of the Omicron variant in recent times. One of the dreadful complications of the SARS-CoV-2 virus infection is immune-mediated thrombotic thrombocytopenic purpura (iTTP). The incidence of iTTP is approximately three per one million adults per year, based on the data from the Oklahoma Thrombotic Thrombocytopenic Purpura-Hemolytic Uremic Syndrome (TTP-HUS) Registry. The median age for the diagnosis of iTTP is 41 years, with a wide range (9-78 years) [1]. The complete "pentad" of microangiopathic hemolytic anemia (MAHA), thrombocytopenia, fever, acute kidney injury (AKI), and severe neurologic findings is not so common (<5%) now due to early recognition and prompt initiation of treatment.

Thrombotic thrombocytopenic purpura (TTP) is a thrombotic microangiopathy (TMA) caused by decreased activity of a disintegrin and metalloproteinase with a thrombospondin type 1 motif, member 13 (ADAMTS13). Platelet-rich thrombi in small vessels contribute to hemolytic anemia. TTP is a medical emergency that can lead to high mortality if not treated urgently. However, with appropriate treatment, the prognosis of survival is good. The pathophysiology of acquired TTP is due to an autoimmune process that triggers the development of autoantibodies against ADAMTS13, which leads to MAHA. TTP has been reported to be due to hereditary and abnormal pathological variation of the gene encoding the ADAMTS13 protein. The most common class of anti-ADAMTS13 autoantibodies is IgG (IgG4 being the most common) [2]. Plasma exchange with fresh frozen plasma is the mainstay for the management of TTP [3]. There is also evidence that steroids are superior to cyclosporine A when used in combination with plasma exchange in the initial management of iTTP [4]. High doses of steroids (typically methylprednisolone 1,000 milligrams intravenous for three days in patients with very high-risk features followed by oral prednisone of 1 milligram/kilogram/day) are beneficial initially for the management of TTP [5]. Goals of stopping steroid therapy would be appropriate after three to four weeks following a clinical response. Rituximab and caplacizumab are clinically proven drugs in the initial treatment of TTP and during the acute phase of relapse. Clinical trials (TITAN [6] and HERCULES [7]) have demonstrated that caplacizumab, when used as an adjunct to therapeutic plasma exchange and steroids, reduces recovery time (normalization of platelets).

Our case describes a 26-year-old male with no significant past medical history who presented with fever, AKI, severe thrombocytopenia, seizures, and acute hemolytic anemia. He was intubated at an outside facility for status epilepticus prior to arrival. The patient was vaccinated with one dose of the Moderna vaccine seven months before presentation to the hospital. He had completely recovered from a prior COVID-19 infection three weeks before the current hospital visit and was discharged home with a short course of antibiotics after one day's stay at another facility. The purpose of this case report was to broadcast the severity of TTP with a recent COVID-19 infection in a young healthy individual and to highlight the response to individualized management for iTTP that followed an active COVID-19 infection.

## Case presentation

The patient initially developed seizures after playing a video game approximately one day prior to admission. He was evaluated at an outside facility for seizures that were unresponsive to antiepileptics and was subsequently transferred to our facility for further management.

**Figure 1:**
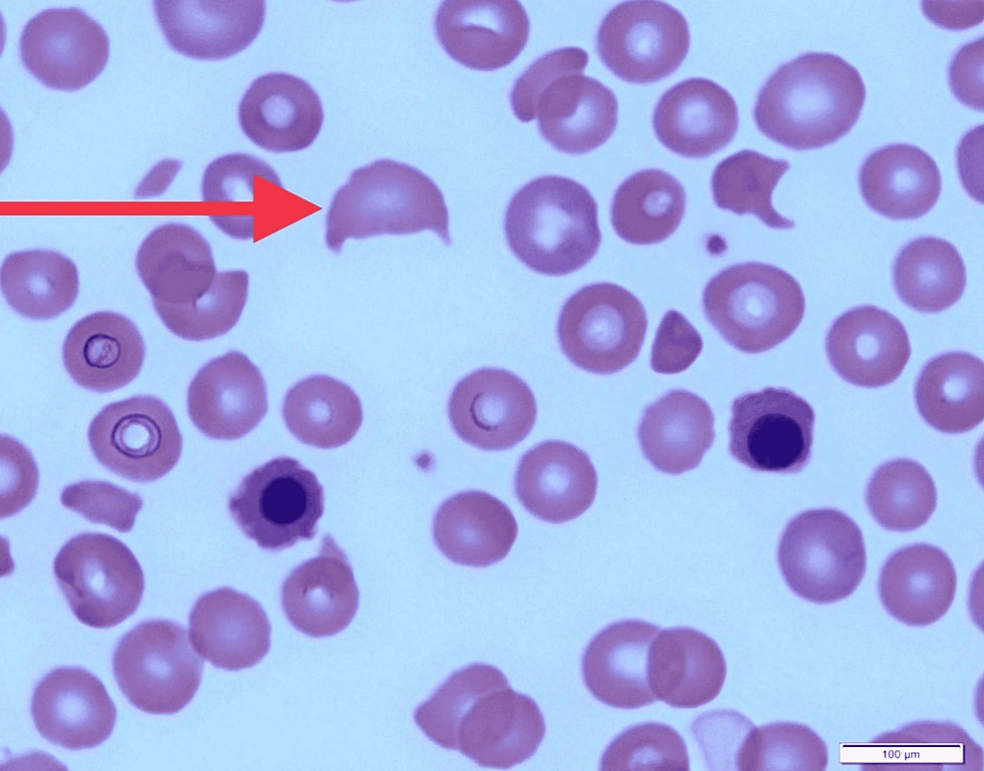
Peripheral blood smear with Leishman stain showing anisopoikilocytosis, nucleated RBCs, severe thrombocytopenia, and schistocytes. Image under 1000x magnification.

**Figure 2:**
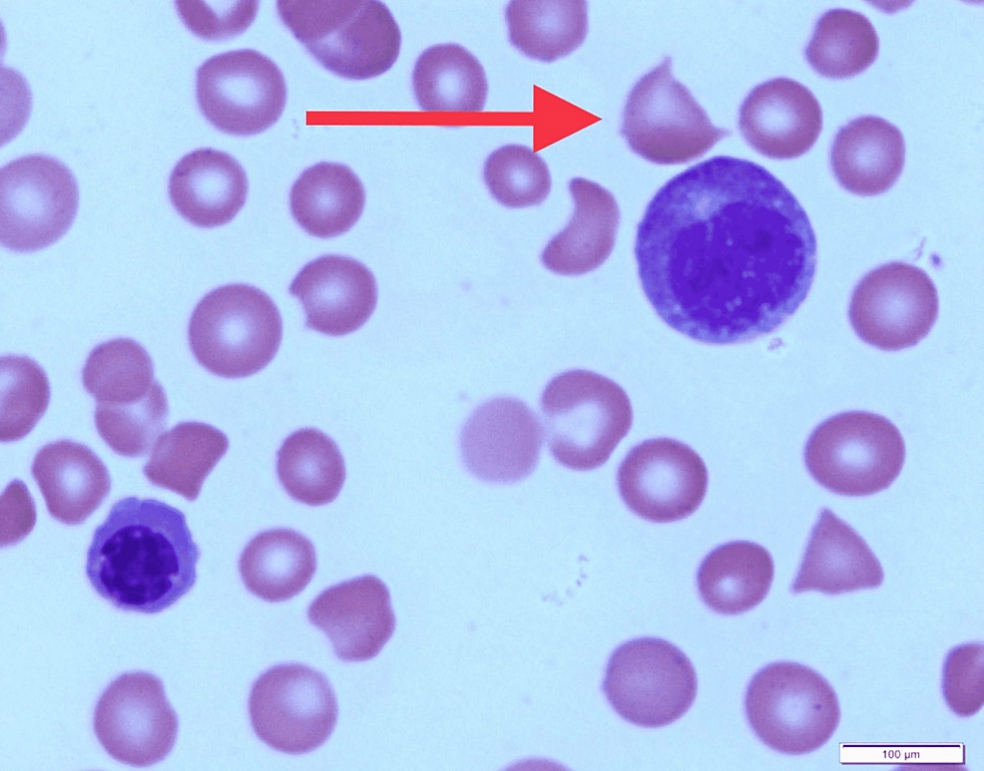
Peripheral blood smear with Leishman stain showing anisopoikilocytosis, nucleated RBCs, severe thrombocytopenia, and schistocytes. Image under 1000x magnification.

**Figure 3:**
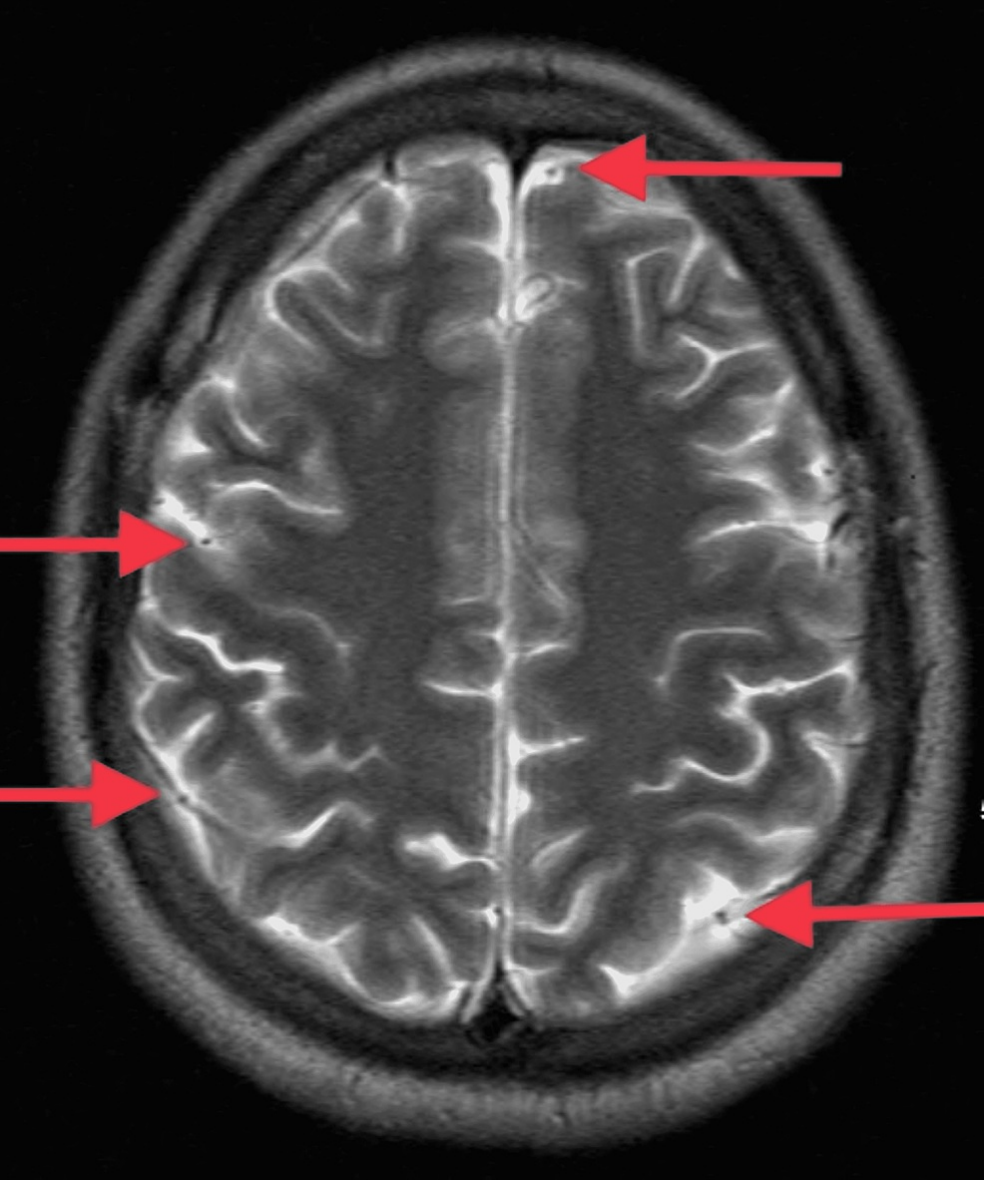
MRI of the brain without contrast at T2-weighted sequence demonstrating "halos" pointed with arrows suggesting microhemorrhages.

**Figure 4:**
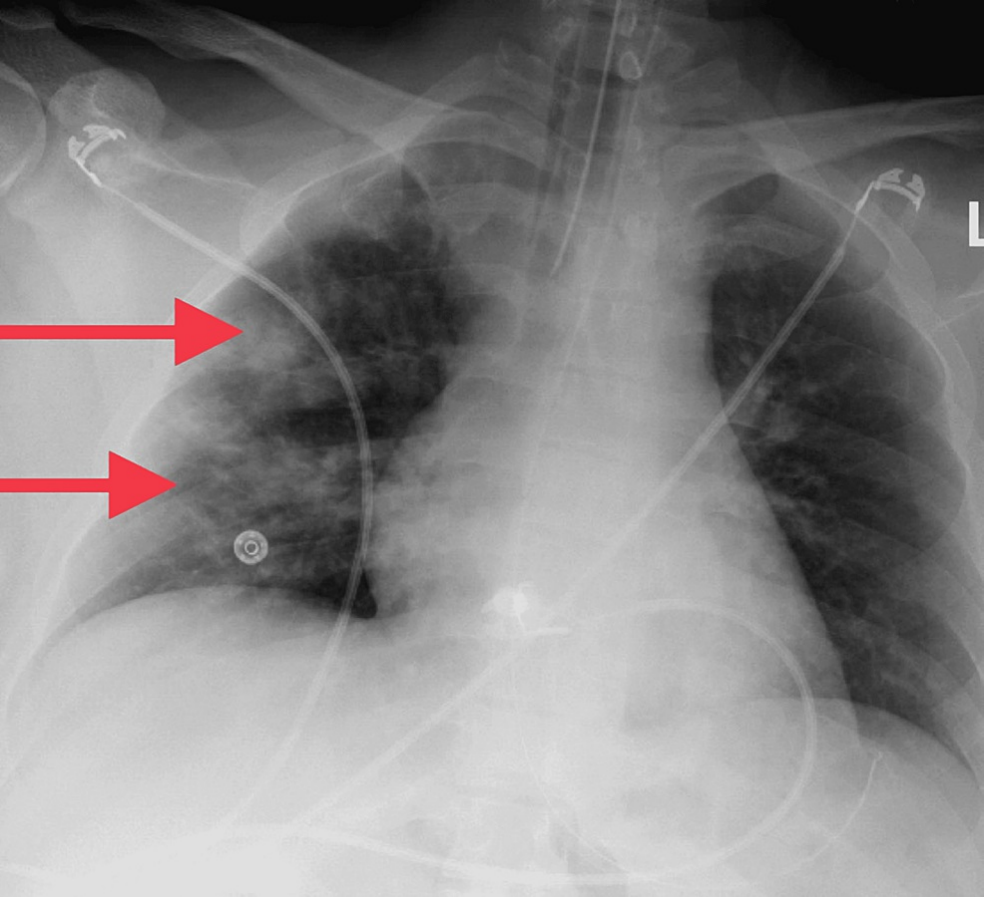
Chest X-ray (anteroposterior view) demonstrating right-sided pulmonary infiltrates as pointed by arrows.

At the time of presentation to our hospital, the patient was intubated, unresponsive, and subsequently transferred to the critical care unit (CCU) due to concerns for status epilepticus. Scleral icterus, multiple petechiae, and hematuria were evident upon physical exam. Laboratory studies revealed severe thrombocytopenia and anemia with hemolysis. Peripheral blood smear showed schistocytes and reticulocytes with an absence of platelets, as shown below in Figures 1, 2. PLASMIC [8] score was 5, which demonstrated an intermediate probability of TTP. CT of the head with contrast performed at another hospital was unremarkable. MRI of the brain without contrast was performed, which showed some “halos” (Figure 3), which were thought to be microbleeds with ongoing MAHA. Chest X-ray demonstrated patchy airspace disease predominantly in the right lung (Figure 4). Clinical picture and schistocytes on peripheral blood smear were suggestive of MAHA that guided us to start plasma exchange and high-dose steroids for a presumed diagnosis of TTP. Due to worsening renal function and oliguria, hemodialysis was initiated in addition to plasma exchange. ADAMTS13 activity was undetectable and ADAMTS13 inhibitor levels were elevated. Rituximab was then started and caplacizumab was added due to high-risk features.

The patient had improvement in encephalopathy by day five; however, lactate dehydrogenase was high and platelet counts were still low after five days of admission. The patient’s mentation improved considerably, and we were able to stop hemodialysis as his renal function normalized. Then plasma exchange was stopped as his platelet counts continued to improve. The patient was discharged home with folic acid supplementation, tapering dose prednisone, and caplacizumab with close follow-up with hematology and oncology with regular monitoring of laboratory studies, as listed in Table 1 below. ADAMTS13 activity became normal after four weeks and platelet counts remained normal. The patient completed the prescribed course of caplacizumab and a tapered dose of steroids and demonstrated a “clinical response” [9] after weeks of active management as described above.

**Table 1:** Laboratory findings monitored with respect to the day of admission and outpatient follow-up visits as listed. LDH: lactate dehydrogenase; PTT: partial thromboplastin time; INR: international normalized ratio; PT: prothrombin time; mg/dL: milligrams per deciliter; IU: international units; BEU: Bethesda units; sec: time in seconds; ADAMTS13: a disintegrin and metalloproteinase with a thrombospondin type 1 motif, member 13.

Labs/day of admission	Reference values with normal limits with respective units	Day 0 - initiated plasmapheresis and glucocorticoids	Day 2	Day 3	Day 9 - initiated daily Cablivi	Day 10 - Initiated weekly Rituxan	Day 17 - Rituxan 2nd dose	Day 24 - Rituxan 3rd dose	Day 31 - Rituxan 4th dose	Day 37 - day of discharge	First outpatient follow-up with hematology (1-week post-discharge)	Second outpatient follow-up with hematology (monthly visit)	Third outpatient follow-up with hematology (monthly visit)
Creatinine	0.7-1.3 mg/dL	1.7	4.68	5.58	6.71	7.41	6.44	4.08	2.96	1.63	2.0	0.9	1
Hemoglobin	13.2-16.6 g/dL	6.4	8.8	9.2	10	9	8.2	6.3	7	9.3	13.4	11.5	12.2
Hematocrit	41-50%	18.3	25.9	26.8	29.9	27.3	24.2	18.2	20.4	26.7	41	34.4	36.4
Platelets	150,000-450,000/uL	15	30	48	116	111	90	70	70	137	199	154	161
Total bilirubin	0.1-1.2 mg/dL	-	-	1.2	2	1.4	0.8	-	-	1.0	1.3	0.9	0.9
ADAMTS13 activity	0.65-1.78 IU/mL	-	-	<0.03	-	-	-	0.9	-	-	-	-	-
ADAMTS13 inhibitor levels	Undetectable BEU	-	-	4	-	-	-	Undetectable	-	-	-	-	-
Reticulocyte count	0.5-2.5%	13.16	8.76	9.21	-	-	-	-	1.54	1.46	-	2.5	
Haptoglobin	41-165 mg/dL	37.7	-	-	-	-	-	-	-	<30	-	-	-
LDH	105-333 IU/L	2836	875	587	596	372	300	184	237	299	440	303	326
PTT	25-35 sec	37.7	-	-	-	-	-	-	30.4		-	-	-
INR ratio	1.1 or below	1.2	1.1	1.1	-	-	-	-	1	0.9	-	-	-
PT (sec)	11-13.5 sec	14.4	13.8	13.9	-	-	-	-	12.7	11.1	-	-	-
Coombs IgG	Negative	-	-	-	Negative	Negative	Positive	-	-	-	-	-	-
Coombs C3	Negative	-	-	-	-	Negative	Negative	-	-	-	-	-	-

## Discussion

COVID-19 has been notorious for its pulmonary manifestations throughout the pandemic. The involvement of various other organ systems, including gastrointestinal, immunological, cardiovascular, neurological, hematological, and psychological manifestations, has been widely recognized. Even with the earliest documented cases of respiratory failure secondary to COVID-19 pneumonia, the COVID-19 virus was known to have a hematological association and was associated with a prothrombotic state. In this case report, we have described a case of TTP, which we believe was sequelae of a recent COVID-19 infection. Although COVID-19 infection-related TTP was documented before, appropriate evidence-based management has not been established yet. We demonstrate a guideline-based modality that was employed for successful treatment. We want to highlight the successful treatment with caplacizumab, rituximab, and multiple cycles of plasmapheresis. Although the pathophysiology of COVID-19-associated TTP is not well understood, it is quite evident that it is related to its primary prothrombotic state and possible consumption of coagulation factors eventually resulting in an increased level of ADAMTS13 inhibitors and an almost undetectable level of ADAMTS13, subsequently leading to MAHA.

In this case, rituximab was employed, which primarily acts on B cells that produce antibodies toward ADAMTS13 and would sufficiently decrease the number of overall circulating antibodies. Similarly, caplacizumab is a von Willebrand factor (VWF) immunoglobulin that has the capacity of inhibiting the interaction between multimers and platelets glycoproteins. This therapy in combination with plasmapheresis was successful in treating TTP, secondary to decreasing the antibodies against ADAMTS13 and at the same time increasing ADAMTS13. The role of plasmapheresis is critical, especially in the setting of worsening renal function associated with TTP.

In our patient, this was successfully treated with a risk-stratified multimodal approach. Platelet counts improved significantly on the ninth day with the use of caplacizumab, as would be expected since caplacizumab works on decreasing the interaction between multimers and platelets glycoproteins. Although this multimodal approach was successful in our patient’s treatment, further research is required to analyze and understand the mechanism and pathophysiology by which COVID-19 infection causes TTP, which would help establish standardized directed therapy in the future.

## Conclusions

Treatment of immune-related TTP with multiorgan involvement can be challenging and requires a multimodal treatment approach. Although plasmapheresis with rituximab and caplacizumab can help improve outcomes as seen in the described case presentation, a different approach is needed when analyzing individual patients with this rare presentation, as molecular pathophysiology of COVID-19’s association with severe TTP and multiorgan failure is not well established. There is also a need to identify future cases, as there have been myths associated with the COVID-19 vaccine and its adverse side effects and complications, to clearly ascertain TTP’s association with active infection and not immunization alone. With the identification of more cases in the future, guideline-directed medical therapy can be established for the treatment and management of these cases.
